# The effect of inter- and intra-layer delay time on TPU parts fabricated by laser powder bed fusion

**DOI:** 10.1007/s40964-024-00933-1

**Published:** 2025-01-09

**Authors:** Samuel Connor, Ruth Goodridge, Ian Maskery

**Affiliations:** Centre for Additive Manufacturing, School of Engineering, The University of Nottingham, Jubilee Campus, Nottingham, NG7 2GX UK

**Keywords:** Powder bed fusion, Laser sinteing, Delay time, TPU, Build packing

## Abstract

In polymer laser powder bed fusion (PBF-LB-P) techniques, such as laser sintering, the time between scanning a given point in one layer and the same *x-y* point in the next layer is known as the ‘inter-layer delay time’. Multiple parts are normally fabricated in a PBF-LB-P build for efficiency; however, this leads to variation in the inter-layer delay time for individual parts; in this study, we present a specific investigation using a commercially available thermoplastic polyurethane (TPU). Multiple part layouts were used and the resulting parts were subject to tensile testing and fracture surface analysis. The results demonstrate that an increase in inter-layer delay time can lead to a significant reduction in mechanical properties. Fabricating specimens in groups of 5 led to a 10% reduction in ultimate tensile strength, 30% reduction in extension at break, and 15$\%$ reduction in Young’s modulus compared to specimens fabricated individually. Fractography suggests this is due to decreased inter-layer bonding and an increase in defects. This has significant implications for the production of multiple parts in a build where consistent mechanical properties are critical. Based on our understanding of this detrimental effect, we put forward a novel build packing approach for PBF-LB-P, based on scanning area equivalence rather than the conventional time minimisation, to mitigate against it.

## Introduction

In polymer laser powder bed fusion (PBF-LB-P) a laser is used to consolidate polymer particles to create 3D parts layer-by-layer [1]. Initially developed for rapid prototyping, PBF-LB-P is now used to create a wide range of end-use products [2] from bicycle helmets [3] to biomedical devices [4], and aerospace parts [5]. These applications take advantage of the general design freedoms offered by AM, as well as those specific to PBF-LB-P. For example, the powder bed provides support to overhangs and isolated regions during fabrication, removing the need for support structures which would otherwise have to be removed post-build. Due to the lack of support structures, many parts can be fabricated in a single build. However, whilst advantageous, the ability to fabricate multiple parts per build is also the source of the ‘delay time’ effect.

The notion of the time between laser scans affecting mechanical properties was established by Williams and Deckard, and Jain et al. [6, 7]. However, most previous work concerns successive laser scans within the same layer, resulting from scan path overlap. Significantly less work has been undertaken to understand the effect of increasing time between laser scans of the same *x-y* point in successive layers. This was noted by Goodridge et al. [8], who reported a significant variation in the magnitude of the effect for different materials. For clarification, we hereby term the two factors ‘pulse delay time’ and ‘inter-layer delay time’, respectively.

Goodridge et al. examined the inter-layer delay time effect in polyamide-12 (PA12), finding that it exhibited no significant change in tensile properties when fabricating one specimen per layer or five specimens per layer [8]. The authors also investigated two elastomeric materials, one of which also exhibited no significant inter-layer delay time effect, while the UTS, EAB, and E of the second elastomer each decreased by $~30\%$. Based on this, they proposed that the inter-layer delay time effect has a strong material dependence. This is one of the key motivations for the current work. The effect of inter-layer delay time on PA12 has been shown to be minimal and, as PA12 remains the most widely used material in PBF-LB-P [9], no further investigation has been performed. However, as new material families are becoming available for PBF-LB-P [10], it is vital to establish the existence of material dependence and potential routes to mitigate the negative effects of inter-layer delay time.

There has not so far been a study which has quantified the inter-layer delay time effect while accounting for the numerous other effects present in PBF-LB-P. This paper builds on the prior work by isolating the inter-layer delay time effect in a TPU using multiple part layouts, with the delay controlled by changing the number of parts per layer. Tensile testing was performed to determine the influence of the inter-layer delay time on the resulting mechanical properties. Finally, the cause of the inter-layer delay time effect, and methods to mitigate against it, have been investigated, through the use of SEM fractography and the development of a novel packing algorithm.

## Methods

### Specimen fabrication

**Fig. 1:**
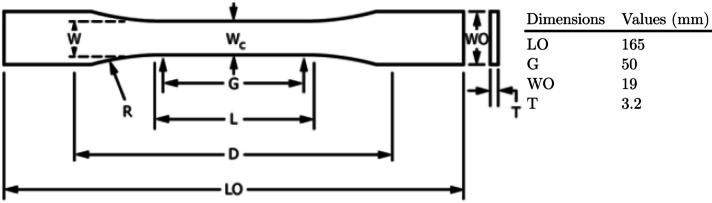
ASTM D638 specimen and key dimensions

To investigate the inter-layer delay time effect, tensile specimens conforming to ASTM D638 [11] were created from EOS-1301 TPU powder. The powder feedstock was purchased from Electro Optical Systems (EOS), Germany. The manufacturer’s datasheet [12] gives the part density, melting temperature and elastic modulus for this material as 1.11 g/cm$^3$, 138 $^\circ$C and 60 MPa, respectively. All specimens were fabricated using new, unrecycled powder from the same batch on an EOS P100 PBF-LB-P workstation. The specimen geometry and key dimensions are shown in Figure 1.

All specimens were fabricated using the same parameters and the same pre- and post-build processes. This included the time between fabrication and testing, as this is known to affect the mechanical properties [13]. No parts aside from those in the inter-layer delay time experiment were included in the builds. TPU feedstock powder was loaded into the EOS workstation with the heaters set to 50 $^\circ$C, and left to dry overnight to remove residual moisture, before the preheating cycle was started. The workstation was preheated for three hours to ensure even heating. After laser processing, the part cake was allowed to cool overnight in the machine and the parts were removed the following morning.

**Table 1:** PBF-LB-P parameters used for processing TPU

Laser power (W)	14
Scan speed (mm/s)	2500
Hatch spacing (mm)	0.25
Bed temperature ($^\circ$C)	105
Scan strategy	Double scan

Table 1 provides the process parameters used to create the TPU tensile specimens. Prior experimentation showed that these parameters consistently led to good part density and shape definition. The material dependent scaling values were set to 1.8% in $x-y$ and 1.5% in *z*. A double scanning strategy was used. The specimens were scanned in both *x* and *y* in each layer. The scan path was bidirectional hatch for each scanning direction.

The EOS P100 workstation does not provide the option to set the inter-layer delay time manually. However, inter-layer delay time is dependent upon the area to be scanned in each layer. Equation (1a) shows how the inter-layer delay time, $T_D$, is defined in this study, being equal to the time taken to scan each layer, $T_S$, added to $T_{\alpha }$ which includes the powder recoating time $T_R$, the computing time $T_{Comp}$, and any other machine-specific delays such as the time taken to reset the laser position.

1a $$\begin{aligned} T_{D}&= T_{S} + T_{\alpha } \end{aligned}$$

1b $$\begin{aligned} T_{\alpha }&= T_{R} + T_{Comp} + ... \end{aligned}$$

1c $$\begin{aligned}&\therefore\,\, T_{D} \propto T_{S} \end{aligned}$$

**Fig. 2:**
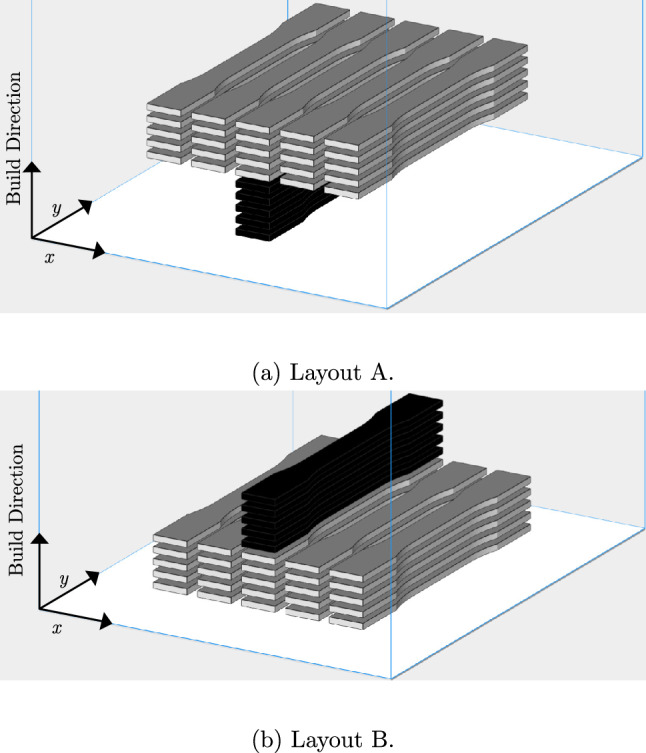
Part layouts to examine the inter-layer delay time effect. 5PPL (parts-per-layer) specimens are shown in grey; the 1PPL in black

For each layer, $T_{\alpha }$ is constant as it depends entirely on machine-specific delays, while $T_{S}$ can be controlled by changing the scanned area. As such, $T_{D}$ can be controlled via $T_{S}$. Figure 2 shows the build layouts used in this work, consisting of single part-per-layer (1PPL) specimens and five part-per-layer (5PPL) specimens. From Eq. (1a) we would expect a slightly less than five-fold increase in the inter-layer delay time between the 1PPL and 5PPL groups, due to the increased scanning area. These layouts help to isolate the inter-layer delay time effect from other effects such as the relative height of each part in the build chamber.

ASTM D638 [11] type 1 specimens were created for tensile testing. Dimension measurements were taken from each specimen to determine the fabrication accuracy, and the mass of each specimen was recorded. The deviation of the specimen geometries from the nominal design dimensions was used to calculate an expansion factor which, combined with the estimated volume from CAD software, provided mass density values for further comparison.

### Characterisation

Tensile testing was carried out using an Instron 5969 universal testing machine, following ASTM D638 [11]. As allowed by that standard, the crosshead speed was 500 mm/min. Load and extension data were collected and mechanical properties, including ultimate tensile strength (UTS), extension at break (EAB) and Young’s modulus (E), were calculated. For each specimen, the measured dimensions were used to calculate the cross sectional area when performing the stress calculations.

The tensile test results were analysed in two ways; first comparing the 5PPL and 1PPL groups, then comparing the variation across rows and columns within the 5PPL groups. This allows for the identification of intra- and inter-layer delay time effects, respectively. For the comparison between the 1PPL and 5PPL specimens, only the central column of 5PPL specimens was used, as this eliminates horizontal position as a variable.

To examine variation within the 5PPL group, the position of each specimen was denoted by their row (*R*) and column (*C*), and an equation of the form

2 $$\begin{aligned} M = \alpha C + \beta R + M_0 \end{aligned}$$

was fitted to the tensile test data. *M* represents any of the mechanical properties determined from the test data (UTS, EAB or E). The gradients $\alpha$ and $\beta$, and their associated uncertainties arising from the fits, were used to identify sensitivity of the mechanical properties to row and column position in the 5PPL group. $M_0$ is an arbitrary fit constant. The naming convention for the 5PPL samples is based on layout A, such that the top left 5PPL sample is A1, and the bottom right sample is E5. This naming scheme is useful for the interpretation of Fig. 4.

Imaging of fracture surfaces was performed using a JEOL JSM IT-200 scanning electron microscope (SEM). Imaging was conducted with a beam voltage of 5 kV at a working distance of 10 mm after being sputter-coated with gold and mounted using carbon cement.

## Results

### Inter-layer analysis

Table 2 shows the average dimensions of the fabricated specimens. The uncertainties shown are standard errors, derived from repeated measurements of each specimen. The thickness (corresponding to the *z* dimension, or build direction) was seen to deviate from the nominal value of 3.2 mm. The largest deviations were recorded for specimens in the 1PPL groups, regardless of layout in the build chamber. Despite the thickness variation between the 5PPL and 1PPL specimens, there is no significant difference in mass density for layout A, and just a 2% density difference for layout B. This suggests that density variations across the examined specimens cannot realistically account for significant variations in mechanical properties.

**Table 2:** Dimensions and masses of TPU specimens in layouts A and B

		Thickness (mm)	Mass (g)	Density (g/cm$^3$)
Layout A	5PPL	3.35 ± 0.02	9.80 ± 0.06	1.124 ± 0.008
	1PPL	3.68 ± 0.01	10.87 ± 0.04	1.125 ± 0.002
Layout B	5PPL	3.28 ± 0.02	9.65 ± 0.04	1.123 ± 0.003
	1PPL	3.65 ± 0.03	11.01 ± 0.06	1.151 ± 0.002

Table 3 shows the average mechanical properties of the specimens from Sect. 2.1, with standard errors originating from five repeat measurements. The underlying data is shown in the tables in Appendix A. As noted with a $*$ in Table 3, some specimens did not reach failure before the testing machine crosshead displacement limit was reached, so a true extension at break could not be calculated.

**Table 3:** Mechanical properties of TPU specimens in layouts A and B

		UTS (MPa)	EAB (%)	E (MPa)
Layout A	5PPL	9.46 ± 0.13	517 ± 20	35 ± 1
	1PPL	10.40 ± 0.07	$>750$^a^	41 ± 0.4
Layout B	5PPL	10.30 ± 0.05	535 ± 11	37 ± 0.2
	1PPL	11.25 ± 0.03	710 ± 13	43 ± 0.2

Representative stress–strain curves are provided in Appendix B

^a^3 of the 5 specimens did not reach failure before the crosshead displacement limit

Going from 1PPL to 5PPL, there is a significant reduction in all mechanical properties, as shown in Table 4. This cannot be explained by variations in density, and it is not related to relative position in the chamber, as this has been accounted for through the use of inverted layouts. The decrease in mechanical properties is therefore assumed to be a result of increased inter-layer delay time. Table 4 states the differences in mechanical properties more clearly, highlighting a decrease of approximately 8% in UTS, >25% in EAB, and 14% in E when build 5PPL compared to 1PPL. The difference in properties between the 1PPL and 5PPL groups is consistent across both examined layouts. This suggests that the inter-layer delay time effect acts independently of other effects, such as height in the build chamber.

**Table 4:** 1PPL-5PPL differences for each layout. Layout A* is a modified version of layout A, as described in Sect. 4.2.1

	$\Delta$UTS (%)	$\Delta$EAB (%)	$\Delta$E (%)
Layout A	9 ± 1	>31	14 ± 1
Layout B	8 ± 1	25 ± 2	14 ± 1
Layout A*	$12\pm 1$	$36\pm 1$	$16\pm 2$

**Fig. 3:**
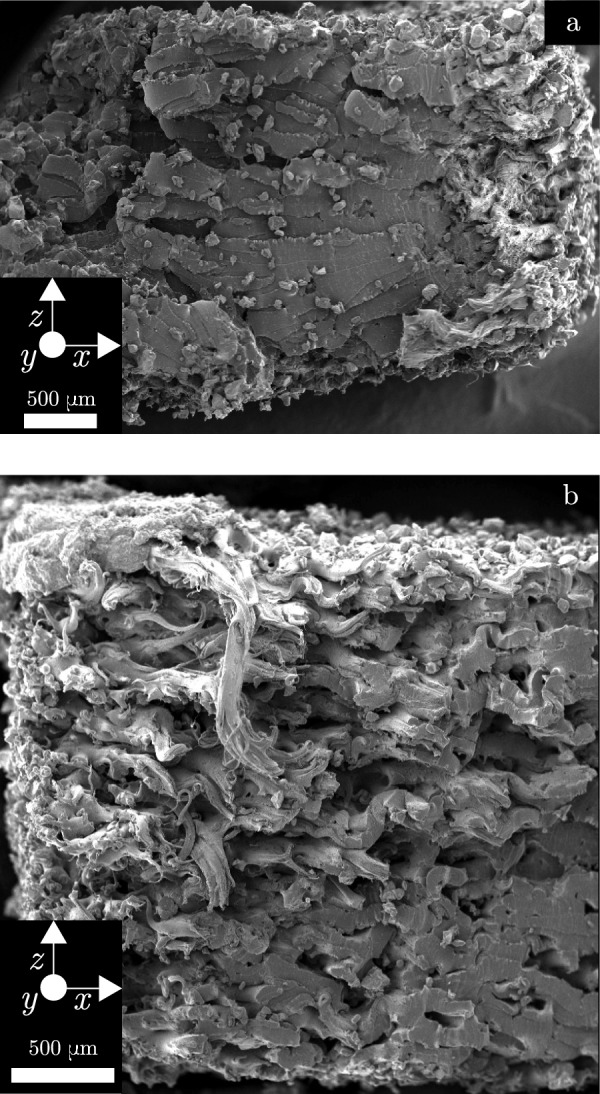
SEM fractography of **a** 1PPL specimen and **b** 5PPL specimen after tensile testing

Figure 3 shows the fracture surfaces from two specimens from layout A. While the 5PPL specimen was taken from the top layer and the 1PPL specimen from the bottom layer of the layout, the results were consistent across positions. The 1PPL specimen in Fig. 3a appears to have a homogeneous fracture morphology, showing signs of ductile failure across the fracture surface. The 5PPL specimen appears to have a heterogeneous fracture surface with some areas exhibiting a more brittle failure mode, which can be seen in the lower-right section of Fig. 3b. The upper region has a more fibrous appearance with the fibres aligned with the loading axis, suggesting ductile failure. There are voids and un-melted powder particles visible in both fracture surfaces. The presence of these features in both 1PPL and 5PPL specimens likely excludes them as the source of the inter-layer delay time effect, given the magnitude of the effect as presented in Table 3; though it should be noted that the 1PPL samples appear to be slightly better consolidated.

### Intra-layer analysis

**Fig. 4:**
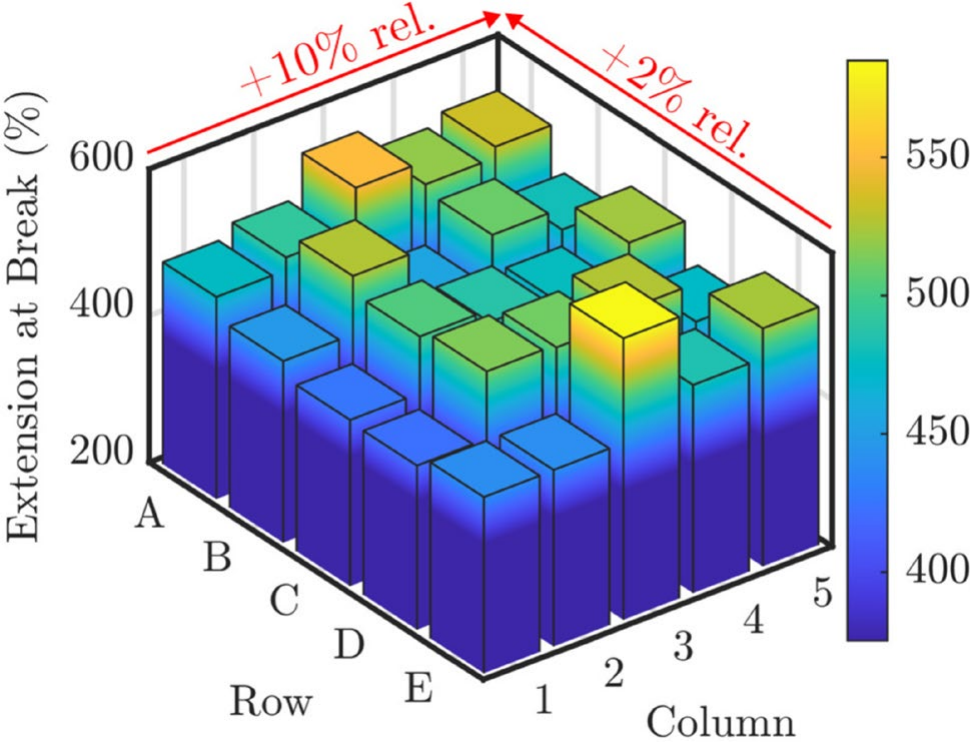
EAB data for layout A. The percentage changes shown here are the relative changes as determined using Eq. (2)

Additional analysis was performed on the 5PPL groups, with the mechanical properties and density being used in Eq. (2) to identify variance according to position within the build chamber. For layouts A and B, the column position was found to have a significant effect on mechanical properties. Figure 4 shows an example of this, where EAB in layout A generally increases with increasing column number. Referring to Eq. (2), for the data shown in Fig. 4 the gradient $\alpha$ is equal to $14\pm 5$ strain $\%$ per column, meaning a specimen in column 5 will generally have an additional 50 percentage points EAB compared to a specimen in column 1. It was found that UTS, EAB and E all increased with column number, but the effects for UTS and E were smaller, with $\alpha$ equal to $0.12 \pm 0.03$ MPa per column number and $0.71 \pm 0.26$ MPa per column number, respectively. This is in keeping with Tables 3 and 4, where EAB is the property most affected by inter-layer delay time. A much smaller correlation was found between row number and mechanical properties, at the limit of numerical significance, suggesting that the vertical position of parts in the build chamber had no effect on mechanical properties.

## Discussion

### Inter- and intra-layer delay time effects

Table 3 shows significant differences in the mechanical properties of the specimens. The 1PPL specimens have larger UTS, EAB and E regardless of layout in the build chamber, with the largest deviation, around 30%, between the 1PPL and 5PPL specimens being for EAB. Additionally, the 5PPL specimens produced in layout B have higher mechanical properties than those from layout A. This could be due to the position in the build volume leading to a lower cooling rate. Josupeit and Schmid [14] showed that the powder cooling rate decreases as the amount of powder increases, arguing that the applied powder acts as insulation. This would lead to slower crystallisation, and therefore better homogeneity, for parts placed higher in the build volume, and hence higher mechanical properties.

In PBF-LB-P, the powder bed temperature is typically set at the melting onset of the material, such that the laser provides enough energy to increase the temperature of the powder above the melting temperature. If the powder has a narrow processing window, defined as the difference between the melting onset and crystallisation onset temperatures, the bed temperature may be too close to the crystallisation temperature, in which case specimens will prematurely crystallise. Nelson et al. [15] found that the bed temperature could decrease by more than 10 $^\circ$C after recoating, which may lead it to decrease below the crystallisation point. However, a distinction between ‘bed temperature’ (typically used to refer to the supporting powder bed) and ‘part temperature’ (the temperature of the area being scanned) should be made here. The cooling of the part to below the crystallisation point, in part due to insufficient bed temperature, is a likely contributor to the inter-layer delay time effect. For the 1PPL specimens, there is less time for the melted material to cool before rescanning. This is assumed to lead to increased crystallinity, and hence less shrinkage, leading to thickness values which deviate further from the nominal value than the 5PPL specimens.

**Fig. 5:**
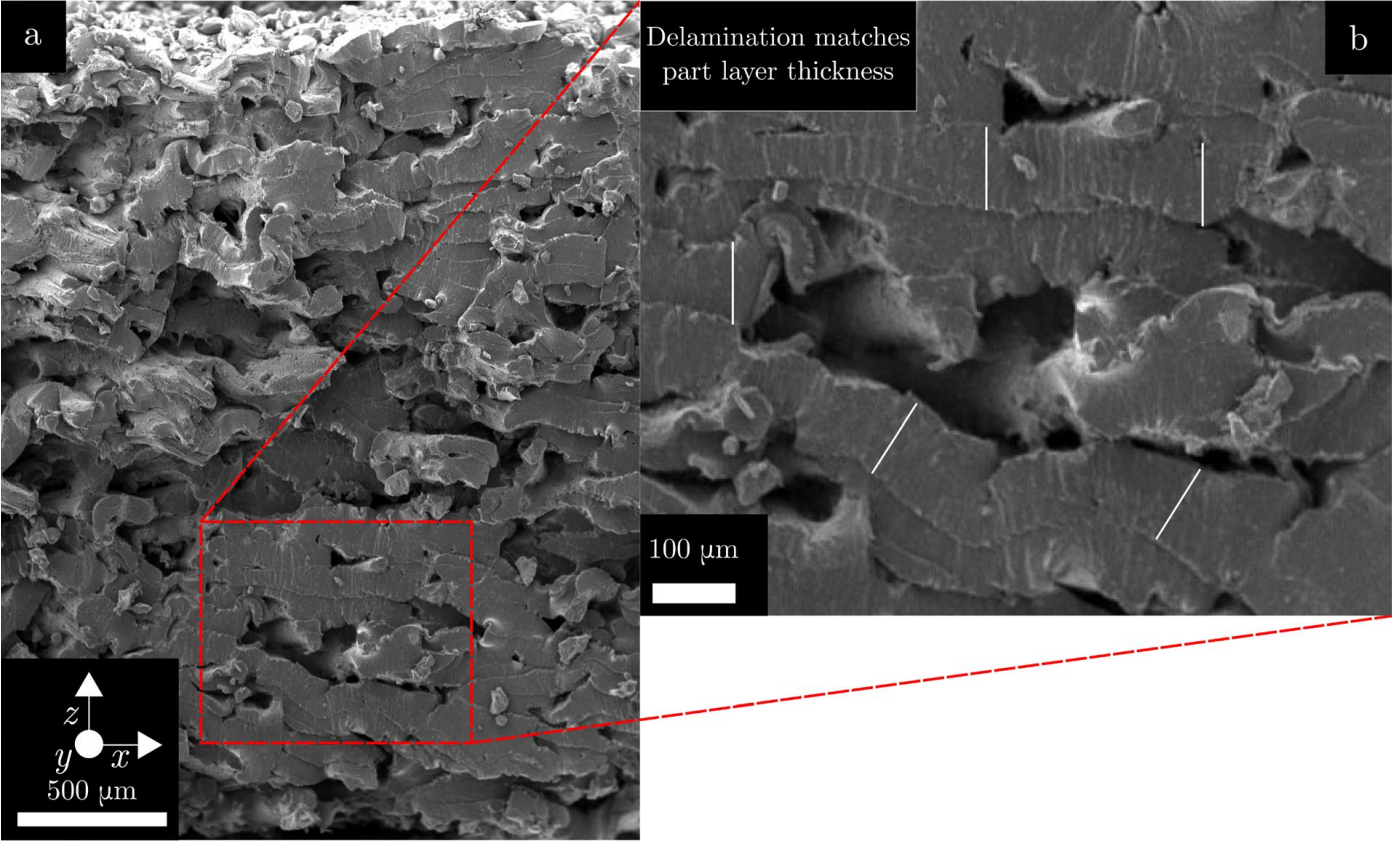
**a** 5PPL sample showing signs of delamination. **b** zoomed image, white lines represent the PBF-LB-P layer thickness

Figure 5 shows clear layers in the fracture surface of a 5PPL specimen. These correspond to the layer thickness of the PBF-LB-P process. This may be due to the increased cooling time for 5PPL specimens before the subsequent laser scan, preventing proper inter-layer adhesion due to increased viscosity in the melt pool. This reduction in inter-layer bonding is consistent with the reduced specimen strength, as given in Table 3 and supports the hypothesis previously put forward that part temperature and inter-layer cooling is the cause of the inter-layer delay time effect.

The effect of specimen orientation on resulting part properties may have some parallels with the inter-layer delay time effect. Changing the orientation of a part may change the cross sectional area present in each layer, and hence change the inter-layer delay time. Kiani et al. [16] investigated the effect of fabricating PA12 parts in two configurations, edgewise and flat, which changes the total area scanned by the laser in each layer, hence indirectly changing the inter-layer delay time. They found that specimens produced in the flat orientation (larger inter-layer delay times) exhibited a 10–15% reduction in UTS and a $~50\%$ reduction in extension at break (EAB). These results also suggest that material ageing may also be a key factor; previous studies [7, 8] using this grade of material (PA2200 from EOS) have not shown any significant effect. However using a mix of 50/50 used/new powder creates a significant inter-layer delay time effect. Xu et al. [17] performed a similar experiment using a TPU material, finding a reduction in UTS of 5% and reduction of 12% in EAB when producing specimens in the flat orientation compared to on their edge.

**Fig. 6:**
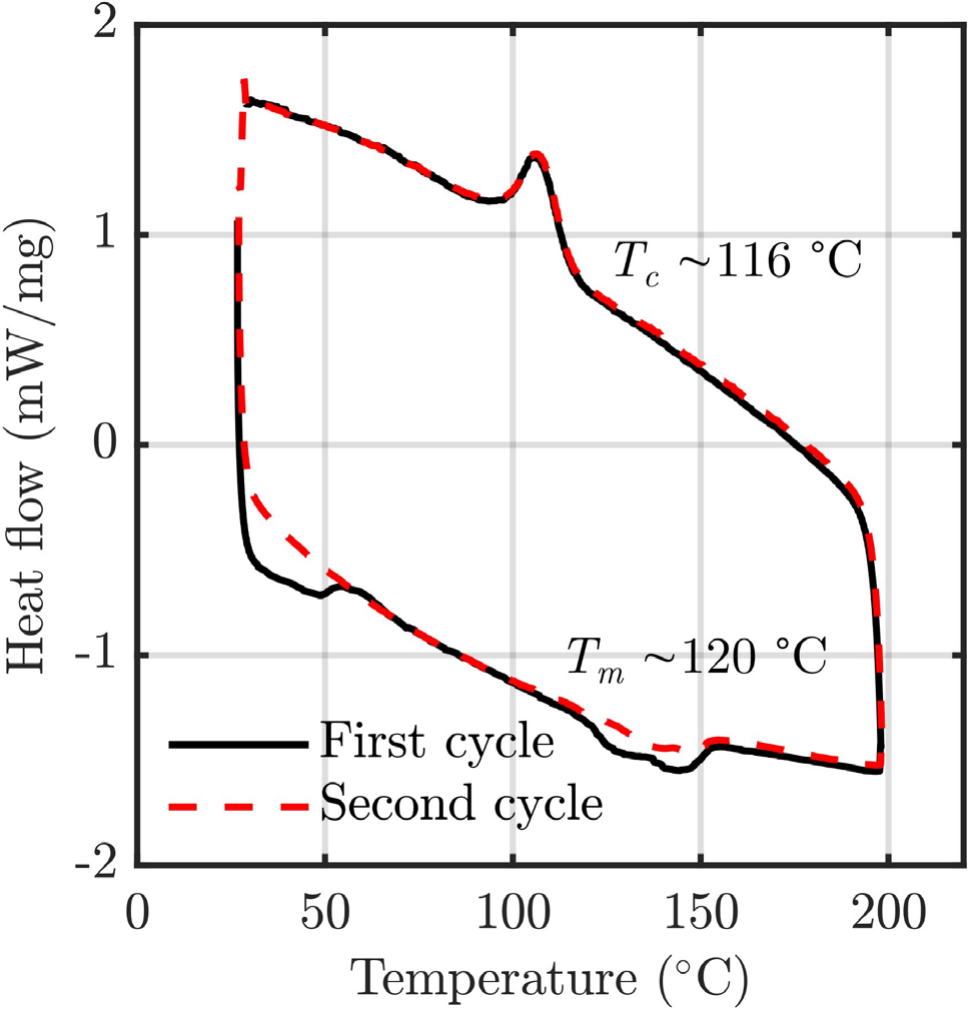
DSC thermograph for TPU material. $T_c$ and $T_m$ are the onset crystallisation and melting temperatures, respectively

Past studies into the inter-layer delay time effect have mostly examined PA12, the most widely used material in PBF-LB-P [9], which has a wide processing window of approximately 30 $^\circ$C [18, 19]. This may explain why studies examining PA12 [7, 8, 20] did not find large inter-layer delay time effects. The DuraForm Flex analysed by Goodridge et al. has a processing window of approximately 40 $^\circ$C [21], as does the WANFAB-PU95AN TPU used by Xu et al. [17]. Neither material showed a significant inter-layer delay time effect. The 1301 TPU used in this work has a process window of approximately 4 $^\circ$C, as shown in Fig. 6. We propose that there is a link between the width of a material’s processing window and its susceptibility to the inter-layer delay time effect. While further investigation is required to establish the exact nature of the relationship, this knowledge can be used to inform design and manufacturing strategies that mitigate against the inter-layer delay time effect.

The intra-layer variation in mechanical properties outlined in Sect. 3.2 appears to be contrary to the variation noted in the literature. Several studies have shown a temperature variation within the build chamber [8, 22, 23], with the highest temperature being achieved in the centre of the chamber; this leads to increased part density in the centre which then reduces towards the corners. But in this work there is no significant change in density across either the rows or columns of the 5PPL specimens. The highest mechanical properties are not found at the centre but the right-hand edge, which cannot be explained by the temperature distribution model presented in the literature. We therefore propose that there is an intra-layer delay time effect arising from the heterogenous cooling experienced by each specimen in the layer. This is because the laser of the EOS P100 workstation scans each layer in the same order, from left to right across the build chamber. This causes the leftmost specimen to be exposed to the build chamber atmosphere for longer than the rightmost, creating a gradient of ‘exposed time’ across the build. The specimens with longer exposed times cool to, or below, the crystallisation temperature before they are recoated, which leads to weaker inter-layer bonding and therefore lower mechanical properties. Since we have only observed this effect in one grade of TPU, we cannot say that this represents a problem for all PBF-LB-P materials. It may be unique to thermoplastics, which often require a bed temperature below the crystallisation temperature of the material to process successfully [24, 25].

### Minimising the inter-layer delay time effect

Given the magnitude of the inter-layer delay time effect reported here, it is important to investigate methods to reduce its impact. If the inter-layer delay time effect is a consequence of the intrinsic thermal characteristics of the material, as hypothesised here, then changes must be made to how the parts are made. Two methods are discussed here: changing the PBF-LB-P process parameters and a novel packing approach.

**Fig. 7:**
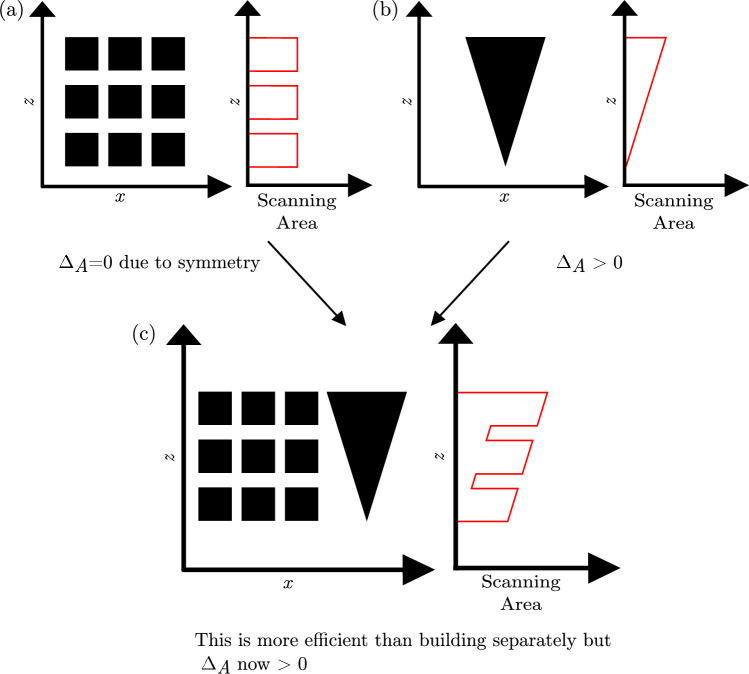
Combining parts to increase packing efficiency in PBF-LB-P may come at the cost of increased inter-layer delay time effect ($\Delta _{A}$)

#### Process modification

Equation (1a) states that the inter-layer delay time is a function of the scanning time, $T_S$, and a machine specific factor $T_{\alpha }$ which includes recoating, etc. So $T_S$ could be modified throughout a particular build, to minimise the inter-layer delay time effect between individual parts. To test this, Layout A (Fig. 2) was recreated, with the 5PPL specimens processed using an increased scanning speed of 3000 mm/s. The laser power was increased to 19.6 W to maintain the same linear energy density. The 1PPL specimens were processed using the same parameters as in Table 1. The resulting specimens were tested using the procedure outlined in Sect. 2.2. These specimens are referred to as Layout A* in Table 4.

No significant reduction in the inter-layer delay time effect was found, despite the increased scan speed for the 5PPL specimens. An explanation for this was found by conducting a short experiment in which some layers of the powder bed were left empty and some contained three tensile specimens. Empty layers, in which there was no area to be scanned by the laser, took approximately 20 s to complete; this is essentially $T_{\alpha }$, a machine constant. Completing a layer containing three specimens took 28 s. So $T_{\alpha }$ contributes significantly to the overall layer time for the EOS P100 workstation. This explains why a $20\%$ increase in scan speed did not significantly decrease the inter-layer delay time effect reported in Table 4; it only led to an  8% decrease in $T_D$ for the three specimens, due to the predominant size of $T_{\alpha }$. For larger scannable areas, increasing scan speed may help to reduce $T_D$ as the significance of $T_{\alpha }$ would be diminished. There is also scope for scanning strategy optimisation to play a role in reducing delay time, as this has been identified as a means of minimising laser scan time for complex geometries [26].

#### Proposed method to reduce the inter-layer delay time effect by part packing

As the inter-layer delay time is proportional to the scanned area in each layer, parts with non-constant cross-section in the build direction will experience a range of inter-layer delay times over the build process, leading to inconsistent mechanical properties within the same part. This is clearly far from ideal for commercial products. To make some progress in this area, we will use a new term, $\Delta _A$, defined as the difference between the maximum and minimum layer area; this can be applied either to individual parts or to an entire build volume of parts. Jain et al. [7] recommend orienting the part to avoid large $\Delta _A$, but depending on part geometry this is not always possible. This is why the packing of parts within the build volume is of crucial importance. Parts with zero $\Delta _A$ can still suffer a inter-layer delay time effect due to other parts in the same build. Conversely, a collection of parts with large $\Delta _A$ could be packed together to minimise the overall $\Delta _A$ of the build. Packing a group of parts in a build volume with this objective is novel, and could potentially solve the problem of mechanical property variation in parts stemming from the inter-layer delay time effect. This would increase the consistency in part properties, while also helping to achieve the best possible part properties by avoiding packing algorithms that focus solely on time- or cost-minimisation.

PBF-LB-P packing efficiency has been the subject of research for many years [27–29], but the effect of $\Delta _{A}$ has not been considered. This can result in part layouts which are volumetrically optimised, and therefore are made with the lowest cost, but which lead to large $\Delta _{A}$. This is illustrated in Fig. 7. The square section parts in Fig. 7a would otherwise have zero $\Delta _{A}$ stemming from their geometry and layout, but the inclusion of other parts (as shown in Fig. 7c) increases the $\Delta _{A}$ of the whole build. In such cases, it may be preferable to reduce the volumetric efficiency in order to achieve lower $\Delta _{A}$. This may increase production costs but help ensure the material properties are homogeneous within each of the parts.

To investigate this new approach to PBF-LB-P packing an algorithm was created based on the Fast Fourier Transform search method of Cui et al. [30]. Our implementation is the same as presented by Cui et al., but we have added new cost functions. A description of our modifications is given here, but for a full specification of the algorithm, including complete mathematical definitions, the reader is directed to the original work [30]. By changing the cost function it is possible to optimise the packing for various metrics. In PBF-LB-P, this is typically a height optimisation due to the direct correlation between build height and fabrication cost. The same approach can be used to minimise $\Delta _{A}$.

When minimising total build height, the dominant contributor to the cost function, $\lambda$, is a cubic height penalisation. There is also a distance term, $\rho$, which penalises placements which are far from the existing parts. When optimising for $\Delta _{A}$, we set $\lambda = Q + \rho$, where *Q* is a term derived from a circular convolution of the area of the new part and the existing parts. The cost function is calculated for every point in the packing volume and the part is placed at the point with the lowest value of $\lambda$.

If no acceptable placement can be found, the part is not placed and the algorithm attempts to place the next one, until it has attempted to place every remaining part. The parts are initially sorted in size order such that the largest (determined from its bounding box) is placed first. Each part is placed in the first acceptable placement, which is known as the first-fit-decreasing method.

**Table 5:** Cost function comparison for somacube part packing—see Fig. 8

Cost function	Height (mm)	$\Delta _A$ (mm$^2$)	Packed parts
Height	30	600	5/7
$\Delta _A$	30	101	6/7

To illustrate this new approach, a packing layout was created using each of the two cost functions, for height and $\Delta _A$. The somacube parts created by Araújo et al. [27] were used as they represent a balance of geometric complexity whilst also having a known solution; the parts can be packed into a cubic volume in numerous configurations. For a representative cubic build volume with side length 30 mm, the results are shown in Table 5. These results demonstrate that $\Delta _A$ can be reduced significantly by changing the cost function. There was no significant increase in computation time for the new cost function.

**Fig. 8:**
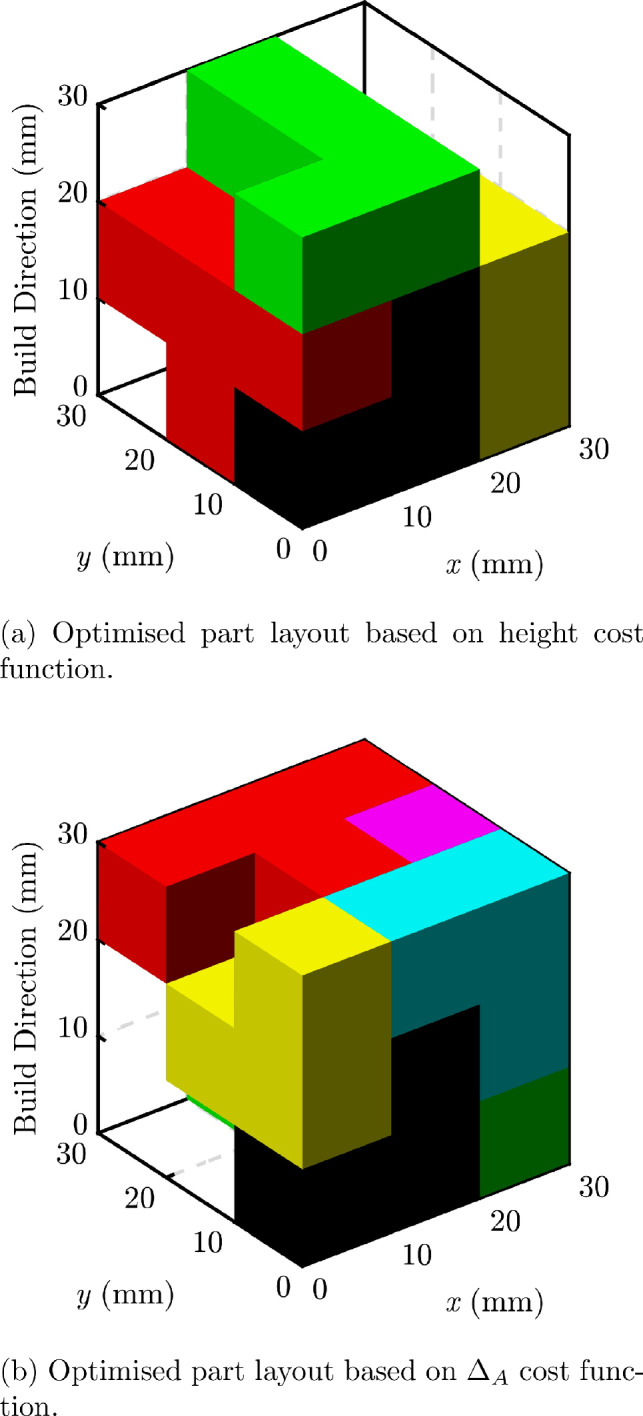
Somacube part layouts created using differing cost functions

Figure 8 shows the two part layouts from Table 5, while also highlighting an important issue. The somacube parts *could* fit together to form a cube with a side length of 30 mm, and therefore no $\Delta _A$, but neither algorithm, using the height or $\Delta _{A}$ cost function, was able to achieve this. This is due to the first-fit decreasing algorithm, which can lead to sub-optimal configurations. It is, however, a classical approach to bin packing [31] and is widely used for PBF-LB-P build optimisation. It is therefore suitable to test our new $\Delta _A$ minimisation method. As noted by Cui et al. [30], a combinatorial/exhaustive search may provide better packing results in general, but at the cost of greater computation time.

**Table 6:** Cost function comparison for Thingi10K part packing—see Fig. 9

Cost function	Height (mm)	$\Delta _A$ (mm$^2$)	Packed parts
Height	86	2197	19/20
$\Delta _{A}$	99	1542	20/20

As a further exploration of this packing approach, a set of more realistic part geometries was obtained from the Thingi10K database [32] and packed using the same cost functions. The packing volume was set to the same aspect ratio as the EOS P100 build chamber used in this work.

**Fig. 9:**
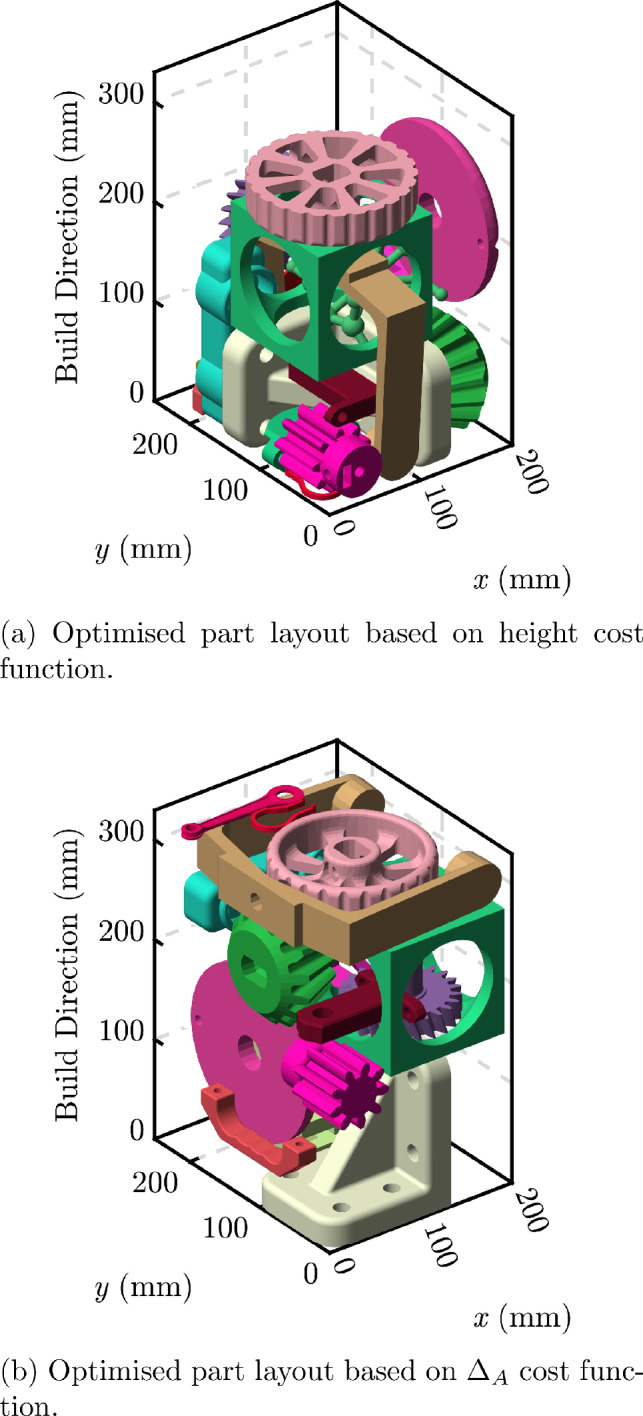
Part layouts created using differing cost functions. The geometries were obtained from the Thingi10K database [32]

Table 6 shows the results of this comparison. The decrease in $\Delta _{A}$ between the two part layouts is about $30\%$ when the $\Delta _{A}$ cost function is used, but this comes at the expense of an additional 13 mm of build height. This is because the $\Delta _{A}$ cost function leads to preferential filling in the *z* direction, while the conventional height cost function attempts to fill the build volume in the *xy* plane first, only increasing the height when the *xy* plane is full. The two layouts from Table 6 are shown in Fig. 9.

While this new packing approach iterates through part positions and orientations to achieve a significant reduction in $\Delta _A$ for the whole build, there are practical reasons why an individual part may instead need to be fabricated in a pre-specified orientation. For example, it is well known that PBF-LB-P parts suffer mechanical anisotropy arising from the layer-by-layer fabrication process [33, 34]. The algorithm currently does not have the capability to favour certain orientations for a given part, but it would be possible to include this feature. By pre-selecting permitted orientations for a given part, or by penalising certain orientations within the cost function, the negative effects of mechanical property anisotropy could be reduced.

## Conclusion

In this work we have attempted to isolate the inter-layer delay time effect for TPU fabricated using PBF-LB-P, showing that it has a significant impact on mechanical properties. This can lead to properties which differ significantly from expected values (e.g., those which may be provided in materials specification) and, more importantly, can yield heterogeneous properties within individual parts and across whole builds. We believe the intrinsic thermal properties of the feedstock powder, chiefly the width of the processing window, is responsible for the inter-layer delay time effect, coupled with the variation in scan times associated with the PBF-LB-P of non-uniform parts. We also note the existence of an intra-layer delay time effect which creates a variation in mechanical properties across a single layer, though it is of a smaller magnitude than the aforementioned inter-layer effect. We present a possible explanation for this phenomenon but further investigation is required, as it could play an important role in optimal build packing for some materials.

To mitigate against the inter-layer delay time effect in PBF-LB-P, we have put forward a new approach for part packing; one which is based on reducing variations in scannable area per layer, rather than conventional height penalisation. We predict this can be quite successful, but note that; (a) it should be validated across a range of part geometries and PBF-LB-P platforms before it can be implemented for proper manufacturing control, and (b) the nature of the material dependence for the inter-layer delay time effect may mean that our approach is not beneficial for all cases, perhaps just a subset of processable materials. On this point, determining relationships between $\Delta _A$ and the resulting variance in mechanical properties should be investigated as a matter of importance. If relationships of this kind can be established, compromises between build height and reduction in mechanical properties would be clearer, and designers would be able to decide if the increase in performance would be worth any additional production costs.

## Figures and Tables

**Fig. 10:**
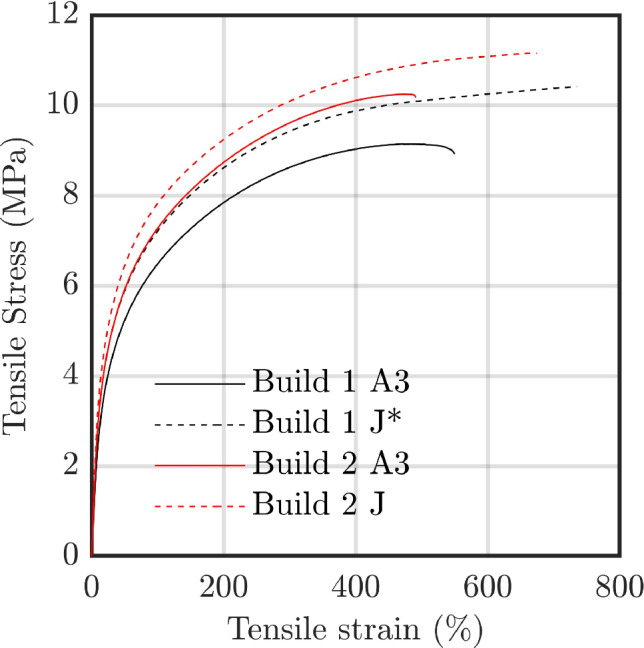
Stress–strain curves for four of the specimens represented in Table 3. Using the same naming scheme as in Appendix A, there are two 5PPL specimens (A3) and two 1PPL specimens (J). Specimen J* did not achieve failure before the crosshead limit was reached

## Data Availability

The authors declare that the data supporting the findings of this study are available within the paper, particularly Tables 7, 8, 9, 10, 11 and 12 in Appendix A.

## Appendix A: Complete mechanical properties for specimens in layouts A and B

In Tables 7, 8, 9, 10, 11 and 12 are given the ultimate tensile strength (UTS), extension at break (EAB) and elastic modulus (E) of each specimen from layouts A and B, as determined from standard tensile tests. In the main body of this paper, the average values derived from this data are presented in Table 3.

**Table 7:** Layout A - UTS per specimen (MPa)

	A	8.98	9.31	9.14	9.27	9.55
	B	9.13	9.52	9.32	9.77	9.71
	C	9.14	9.64	9.35	9.60	9.76
	D	9.01	9.51	9.47	9.62	9.68
	E	9.04	9.41	10.00	9.54	9.57
Row	F	–	–	10.41	–	–
	G	–	–	10.51	–	–
	H	–	–	10.24	–	–
	I	–	–	10.34	–	–
	J	–	–	10.52	–	–
		1	2	3	4	5
		Column

**Table 8:** Layout A - EAB per specimen (%)

	A	475	493	551	519	534
	B	447	527	456	510	480
	C	426	504	487	477	522
	D	423	515	511	526	474
	E	440	440	583	483	524
Row	F	–	–	735	–	–
	G	–	–	752	–	–
	H	–	–	764	–	–
	I	–	–	744	–	–
	J	–	–	763	–	–
		1	2	3	4	5
		Column

**Table 9:** Layout A–E per specimen (MPa)

	A	30.07	31.90	31.59	30.99	31.85
	B	35.42	35.85	35.58	37.62	38.17
	C	34.37	36.00	35.67	36.83	38.50
	D	34.45	35.78	35.92	37.27	38.00
	E	33.61	35.59	38.25	37.11	36.80
Row	F	–	–	37.17	–	–
	G	–	–	42.50	–	–
	H	–	–	42.43	–	–
	I	–	–	42.62	–	–
	J	–	–	41.11	–	–
		1	2	3	4	5
		Column

**Table 10:** Layout B - UTS per specimen (MPa)

	J	–	–	11.20	–	–
	I	–	–	11.16	–	–
	H	–	–	11.29	–	–
	G	–	–	11.28	–	–
	F	–	–	11.29	–	–
Row	E	9.93	10.32	10.40	10.54	10.55
	D	9.98	10.09	10.12	10.39	10.40
	C	9.87	10.14	10.33	10.31	10.54
	B	10.03	10.29	10.41	10.44	10.69
	A	10.32	10.37	10.25	10.37	10.53
		1	2	3	4	5
		Column

**Table 11:** Layout B - EAB per specimen (%)

	J	–	–	682	–	–
	I	–	–	677	–	–
	H	–	–	735	–	–
	G	–	–	736	–	–
	F	–	–	722	–	–
Row	E	461	535	560	551	502
	D	486	503	556	563	521
	C	486	530	540	536	525
	B	474	523	529	540	521
	A	445	503	492	494	509
		1	2	3	4	5
		Column

**Table 12:** Layout B–E per specimen (MPa)

	J	–	–	42.87	–	–
	I	–	–	42.62	–	–
	H	–	–	42.85	–	–
	G	–	–	42.90	–	–
	F	–	–	43.93	–	–
Row	E	35.43	36.91	37.24	38.64	39.07
	D	35.56	36.42	36.82	37.94	38.48
	C	35.68	36.93	37.29	37.73	38.97
	B	35.47	36.81	37.37	37.64	39.90
	A	36.03	37.33	37.42	38.06	39.62
		1	2	3	4	5
		Column				

## Appendix B: Stress–strain curves

See Fig. 10.
